# Integrin-Mediated Adhesion and Chemoresistance of Acute Lymphoblastic Leukemia Cells Residing in the Bone Marrow or the Central Nervous System

**DOI:** 10.3389/fonc.2020.00775

**Published:** 2020-05-22

**Authors:** Bibi Fatima Syed Shah Scharff, Signe Modvig, Hanne Vibeke Marquart, Claus Christensen

**Affiliations:** Department of Clinical Immunology, Copenhagen University Hospital Rigshospitalet, Copenhagen, Denmark

**Keywords:** acute lymphoblastic leukemia, integrins, chemoresistance, CNS, bone marrow

## Abstract

Acute Lymphoblastic Leukemia (ALL) is the most common cancer in childhood. Despite a significantly improved prognosis over the last decade with a 5-years survival rate of ~90%, treatment-related morbidity remains substantial and relapse occurs in 10–15% of patients (1). The most common site of relapse is the bone marrow, but early colonization and subsequent reoccurrence of the disease in the central nervous system (CNS) also occurs. Integrins are a family of cell surface molecules with a longstanding history in cancer cell adherence, migration and metastasis. In chronic lymphoblastic leukemia (CLL), the VLA-4 integrin has been acknowledged as a prognostic marker and mounting evidence indicates that this and other integrins may also play a role in acute leukemia, including ALL. Importantly, integrins engage in anti-apoptotic signaling when binding extracellular molecules that are enriched in the bone marrow and CNS microenvironments. Here, we review the current evidence for a role of integrins in the adherence of ALL cells within the bone marrow and their colonization of the CNS, with particular emphasis on mechanisms adding to cancer cell survival and chemoresistance.

## Introduction

Integrins comprise a family of heterodimeric cell adhesion receptors, each composed of one alpha and one beta subunit. In hematopoietic and epithelial cells, integrin adhesion to stromal cells or extracellular matrix (ECM) components induces signaling essential for cell survival, proliferation and migration. Integrins are also vital to cancer cells, that exploit the integrins to favor their own survival, invasion and migration within tissues, endothelial cell binding, extravasation and metastatic colonization of distal organs (2). In hematological cancers, expression of integrin α7 was shown to be associated with acute myelogenous leukemia (AML) with granulocytic sarcoma (3) and integrin α4:β1 (VLA-4) is an independent prognostic factor in chronic lymphoblastic leukemia (CLL) (4) and has been associated with chemoresistance in CLL, multiple myeloma, AML and acute lymphoblastic leukemia (ALL) (5–7). Thus, integrin-mediated chemoresistance is perceived as a form of adhesion-mediated drug resistance (CAM-DR) (8).

ALL is a malignant disorder of lymphoid progenitor B- or T-cells, representing the most common form of pediatric cancer (patients <15 years of age) (9). ALL is largely a bone marrow (BM) disease and thus diagnosis and treatment stratification using disease monitoring by minimal residual disease (MRD) (10), are done optimally in BM samples. BM constitutes the most frequent site of relapse (11) and has been proposed as a protective niche for ALL (12). In addition, ALL has a marked tendency to disseminate to the central nervous system (CNS) and survive therapy. At the time of diagnosis, 8–13% of ALL patients have measurable leukemic blasts within the CNS (13–15) and despite of the CNS prophylaxis in current treatment protocols, 10–30% of relapses involve the CNS (16–18).

At present, several integrins have been proposed as likely contributors to CAM-DR in ALL and different routes of dissemination from BM to the CNS have been suggested (19, 20). Here, we review the current evidence linking individual integrins to the adherence and chemoresistance of ALL cells within the BM and their dissemination to CNS.

## Integrin Structure and Signaling

In humans, 18 alpha subunits and 8 beta subunits are known, which assemble into 24 different heterodimers. Of these, only half have been identified in immune cells (21, 22). Each integrin consists of a large ectodomain responsible for ligand binding, a transmembrane domain, and an intracellular domain making contacts with the cytoskeleton. Integrins differ considerably with respect to ligand specificity. For example, α6:β1 only binds laminin (23) whereas α4:β1 (VLA-4) can bind the ECM molecules fibronectin, thrombospondin, and osteopontin as well as the cell surface molecules vascular cell adhesion molecule 1 (VCAM-1) and mucosal addressin cell adhesion molecule 1 (MadCAM-1) (21, 24). Today, integrin subunits may be referred to according to the CD (Cluster of Differentiation) nomenclature and integrin dimers may be named according to the scheme α: β or as very late antigens (VLA) in the case of dimers containing β1. Previous reviews by Bertoni et al. and Humphries et al. serve as convenient sources of information on integrin nomenclature and binding partners (21, 24).

Integrins adopt different conformations with distinct ligand-binding affinities. The shift to the extended-open conformation allows for high-affinity ligand binding and constitutes integrin activation (25). This may be achieved via “inside-out” signaling, i.e., intracellular signals received from other receptors or tensile forces acting on the integrin heterodimers through their cytoskeletal connections. Alternatively, activation may arise from “outside-in” signaling through ligand-binding or mechanical forces (26), conferred by e.g., blood- or cerebrospinal fluid (CSF) flow. The latter may induce so-called catch-bonds, which prolongs bond lifetime and allows for more stable adhesion. It plays a role in αL:β2 integrin-mediated adhesion to ICAM-1 during leukocyte arrest on the inner side of inflamed blood vessels (27–29).

When binding ECM molecules, integrins initiate reorganization of these molecules and undergo clustering on the plasma membrane. In the case of α5:β1 binding to fibronectin, clustering of integrins leads to the assembly of fibronectin molecules into larger insoluble fibrils (30). Coinciding with the clustering, multiprotein complexes form on the cytoplasmic side, which serve as a hub for outside-in signaling (31). In general, outside-in signaling commences with recruitment and activation of the non-receptor tyrosine kinases focal adhesion kinase (FAK) and the Src kinase, or alternatively the FAK-related kinase PYK2 and the spleen tyrosine kinase (SYK) (32, 33). Subsequently, these kinases activate multiple pathways involving MEK-MAPK/ERK, PI3K-AKT, JAK-STAT, mTOR, and NFkB proteins (34) thereby affecting a range of cell fate decisions including cell cycle progression (35, 36) and survival vs. apoptosis (37, 38). Interestingly, integrin dynamics involves a continuous recycling of transmembrane integrins to the cytoplasm and FAK kinase remains active when associated with integrins in endosomal membranes. This mechanism contributes to the survival of metastasizing breast cancer cells (39) and could explain how integrins mediate survival signaling also in circulating leukemic blasts.

The ability of outside-in signaling to suppress the apoptotic response is of key importance in integrin-mediated chemoresistance. Many of the agents used as first-line cancer therapeutics, incl. the ALL induction therapy drugs doxorubicine/adriamycin and vincristine, are strong inducers of DNA damage, causing cancer cell elimination by triggering apoptosis (40, 41). Across different cancer types, MAPK/ERK and PI3K-AKT signaling have been shown to increase drug resistance via altered expression of Bcl-2 family proteins (42, 43) or increased activity of the ATP-binding cassette C 1 (ABCC1) transporter/multidrug resistance-associated protein 1 (MDR1) (44, 45). In accordance, the binding of α2:β1 integrin to collagen I has been shown to promote resistance to doxorubicine in Jurkat T-ALL cells by activating ERK and maintaining high levels of the anti-apoptotic Bcl-2 protein Mcl-1 (46) or upregulating ABCC1/MRP-1 (47). Furthermore, activation of integrin β1 by different ECM molecules initiated RAFTK/PYK2-AKT signaling and increased chemoresistance in different leukemic cell lines (48, 49), which was linked to the activation of the ABCC1/MRP-1 (50). In addition, AKT can promote survival signaling along several pathways, e.g., via the mTOR kinase and NFkB and STAT transcription factors (51–55).

The above-mentioned studies show that the interaction between integrins and ECM ligands activates pro-survival pathways that are shared by cancer cells across type. However, some studies suggest more unorthodox mechanisms. Jacamo et al. used an ectopic BM model in mice and described how drug resistance in leukemic cells depended on VLA-4:VCAM1-mediated NFkB activation in stromal cells (56) whereas, Polak et al. showed integrin-dependent cell-cell signaling through tunneling nanotubes between BCP-ALL cells and mesenchymal stromal cells, inducing pro-survival cytokine secretion and prednisolone resistance (57). Another study found that integrin β1 existed in a multi-protein membrane complex together with the potassium channel protein hERG1 (human Ether-à-go-go-Related-Gene 1) and the C-X-C chemokine receptor type 4 (CXCR4) and that stromal cell-mediated chemoresistance in ALL cells was overcome by hERG1 blockade or inhibition of the CXCR4/CXCL12 axis (58). Finally, in various non-leukemic tumor models, β1 integrin binds the proto-oncogenic receptor c-Met whereby it contributes to sustained pro-survival signaling from this receptor in a manner that appears to be independent of ECM ligands (59, 60).

## Adhesion and Chemoresistance of All Within the Bone Marrow

In healthy BM, the localization of hematopoietic stem cells (HSCs) is highly influenced by the microenvironment, including chemokines/cytokines, extracellular matrix proteins and cell surface proteins within both the endosteal and vascular niches. Notably, the endosteal niche is rich in fibronectin and collagen type I (61) whereas laminin and collagen type IV are enriched within the vascular niche (62). Not surprisingly, integrins binding these ECM molecules are expressed on CD34^+^ stem cells and essential for sustaining hematopoiesis (63). *In vivo* experiments including gene ablation and function blocking antibodies indicate that β1-containing integrins are particularly important and emphasize roles of both α4 and α6 (64–67).

In leukemias, cellular proliferation, maturation, adhesion and migration are dysregulated leading to high numbers of premature, malignant cells in the BM as well as in the blood. As for normal hematopoiesis, the homing, survival and egression of leukemic blasts are largely controlled by the microenvironment of the BM and an important role is afforded to the integrin family (68). Table 1 summarizes the most important studies showing integrin-mediated adhesion or chemoresistance in ALL. Overall, studies have pointed to the importance of BM stromal cells in the survival of BCP-ALL cells and the role played by integrins in this interaction (83, 84). In SCID mice, both α4:β1 and α5:β1 have been shown to be important for binding of patient-derived BCP-ALL cells to BM stromal cells (69) and in patients, lower affinity states of α4:β1 on BCP-ALL cells appear to correlate with higher numbers of blasts in circulation, i.e., white blood cell count (WBC) (76). The latter suggests that the retainment of blasts within the BM is largely dictated by α4:β1-mediated adhesion in agreement with studies of hematopoietic stem cells (85, 86).

**Table 1:** Important works demonstrating roles or associations of integrins with chemoresistance, tissue localization or clinical outcome.

**Tissue**	**ALL**	**Integrin(s) involved**	**Key finding**	**References**
BM	BCP-ALL	α4:β1 (VLA4) α5:β1 (VLA5)	High expression correlates with homing to BM in xenograft mouse model	Messinger et al. (69)
	BCP-ALL	α4:β1 (VLA4)	Variant of NALM-6 cell line lacking VLA-4 expression showed reduced BM infiltration in xenograft mouse model	Filshie et al. (70)
	BCP-ALL	β1-integrins	Regulation by SDF-1 and CXCR4 chemokines and its role in BM localization in xenograft mouse model	Bendall et al. (71)
				Shen et al. (72)
	BCP-ALL	α6 (CD49f)	Over-expressed in B-ALL, potential MRD-marker (Clinical BM samples, *N* < 20)	DiGiuseppe et al. (73)
	BCP-ALL	α4 (CD49d)	Natalizumab sensitizes primary ALLs to chemotherapy in xenograft mouse model	Hsieh et al. (74)
	BCP-ALL	α4:β1 (VLA4)	High expression at first relapse is a marker of poor prognosis. (clinical BM samples from patients with relapsed ALL, *N* = 56)	Shalapour et al. (75)
	BCP-ALL	α4:β1 (VLA4)	Lower affinity states correlate with high WBC (clinical samples, *N* = 36)	Blenc et al. (76)
	Ph+BCP-ALL	α5:β1 (VLA4)	Different strategies to interfere with α5 integrin function impair BM engraftment in xenograft mouse model	Hu et al. (77)
	BCP-ALL	α4:β1 (VLA4) αL:β2 (LFA-1)	Increased integrin expression and adhesion to ECM ligands in Sup-B15 cell line overexpressing 5T4 oncofetal antigen. Dissemination studied in xenograft mouse model.	Castro et al. (78)
	BCP-ALL	α4:β1 (VLA4)	Drug resistance in leukemic cells depended on VLA-4:VCAM1-mediated NFkB activation in stromal cells. A xenograft mouse model was used involving extramedullary BM.	Jacamo et al. (56)
	BCP-ALL	α6 (CD49f) α4 (CD49d)	α6 (CD49f) but not α4 (CD49d) associates with persistent MRD (clinical BM and CSF samples, *N* > 100)	Scharff et al. (79)
	T-ALL	α2:β1 (VLA2)	α2:β1/ERK pathway promotes chemoresistance in T-ALL (include studies of chemoresistance in primary T-ALL cultures from patient BM, *N* = 3)	Naci et al. (46)
	T-ALL	β1 (CD29)	Blockade of β1 integrin diminishes leukemic burden in BM (use of xenograft mouse model and primary T-ALL cultures from patients, *N* = 3)	Berrazouane et al. (50)
CNS	BCP-ALL	β2	Increased in BCP-ALL cells capable of brain infiltration, highlights role of CD7/integrin β2 axis (use of xenograft mouse model)	Kondoh et al. (80)
	BCP-ALL	α6 (*ITGA6*)	Downregulated in NALM-6 cells isolated from CNS compared to BM (in a xenograft mouse model)	Gaynes et al. (81)
	BCP-ALL	α6:β1 (VLA6)	Blasts migrate on abluminal side of emissary vessels to invade CNS (use of xenograft mouse model and clinical samples)	Yao et al. (20)
	BCP-ALL/T-ALL	VCAM-1 binding integrins	Me6TREN treatment downregulates VCAM-1, disrupts leukemia-meningeal adhesion and increases drug sensitivity of CNS leukemia (use of xenograft mouse model)	Jonart et al. (82)
	BCP-ALL	α5	Associates with CSF colonization (clinical CSF samples, *N* > 100)	Scharff et al. (79)

*Listed are only clinical studies involving patient samples or preclinical studies involving the study of human leukemic cells in xenograft mouse models. Preclinical studies only focusing on leukemic cell lines in vitro are described in the text*.

Shalapour et al. studied 56 BCP-ALL patients with BM relapse and correlated the expression of α4:β1 in BM samples with clinical outcome. They found that high α4:β1 expression associated with shorter event-free and overall survival (75). Later, Hsieh et al. studied 207 cases of BCP-ALL with detectable MRD at end of induction, confirming a poorer outcome for patients showing high compared to low integrin α4 expression. Furthermore, they provided strong evidence for the chemoprotective function of integrin α4 showing that drug resistance of BCP-ALL *in vivo* was overcome by either *ITGA4* gene knock-out or α4 blockade using the humanized anti-α4 monoclonal antibody natalizumab (74). Natalizumab inhibits both members of the α4 integrin family, α4:β1 and α4:β7, whereas the small non-peptidic molecule inhibitor TBC3486 is 200-fold more potent toward α4:β1 than α4:β7. Using this inhibitor, Hsieh et al. was able to show that the chemoresistant phenotype of ALL was mainly due to α4:β1 (87).

Apart from α4:β1, reports also point to chemoprotective roles of other integrins. In Philadelphia chromosome positive ALL (Ph^+^-ALL), inhibition or knock-down of α5 reduced the leukemic engraftment of BM in NOD/SCID gamma (NSG) mice and reduced the survival of leukemic cells treated with tyrosine kinase inhibitors (77). It is noteworthy, that altered integrin expression patterns have been reported in different types of cancers and in some cases contradictory data for the same integrin are found within the same cancer type (2). A recent study investigating the mRNA and protein surface expression of integrins in ALL failed to confirm the proposed association between MRD and α4/CD49d or α5 mRNA in a large patient group. The strongest association with MRD was instead found for α6/CD49f (79). The reason for these discrepancies is currently unknown. However, integrin regulation is highly complex and what defines integrin function is integrin activation and the resulting ligand affinity of specific integrin heterodimers, rather than the levels of individual integrin subunits *per se*. Hence, studies addressing affinity states of α4:β1, such as the work of Shalapour et al. (75) are likely closer to revealing the association of α4:β1with MRD than studies based entirely on expression profiles, such as the work of Scharff et al. Also, the work of Scharff et al. did not specifically address Ph^+^ ALL, which may be the reason why an association between MRD and α5 was not seen (79).

## Adhesion and Chemoresistance of All Within the CNS

The CNS is an immunologically privileged site to which access is limited by the blood-brain barrier (BBB) and the blood-cerebrospinal fluid barrier (BCSFB). The BBB is composed of the endothelial cell (EC) lining and the EC basement membrane of CNS microvessels plus a second underlying parenchymal basement membrane, which is formed by astrocytes and part of the glia limitans. At the level of CNS capillaries, the glia limitans and the EC basement membrane form a composite layer; whereas at the level of post-capillary venules the two layers are separated by a CSF-filled perivascular space that fuses with the leptomeningeal/subarachnoid space (88). In contrast, the BCSFB exists at the choroid plexus (CP), comprising villous structures bulging from the walls in certain parts of the four ventricles of the brain. The outer layer consists of specialized epithelial cells, known as ependymal cells, that are interconnected by tight junctions and underneath, an extensive network of microcapillaries exists composed of fenestrated endothelial cells (89).

As part of immunosurveillance of the CNS, normal immune cells cross the BCSFB but generally remain in the CSF. In healthy individuals, these immune cells are predominantly central memory CD4^+^ cells (90). However, in response to antigens and ensuing neuroinflammation, proinflammatory cytokines alter the microvessels allowing passage of the BBB at the site of the post-capillary venules by both T cells and myeloid cells (91, 92). Integrins appear to be involved in the trafficking of normal immune cells across the brain barriers during neuroinflammation. Hence, in multiple sclerosis (MS) patients, the α4-inhibitor natalizumab reduced the number of CD4^+^ cells in CSF (93) as well as relapse frequency (94), and in assays that use immune cells from MS patients, natalizumab blocked the transmigration across layers mimicking BBB (93). These studies suggest that α4-containing integrins, e.g., α4:β1 or α4:β7, are involved in the transmigration process across the BBB and/or BCSFB. With respect to crossing the BCSFB, VCAM-1 binding on the basolateral side is less likely, as it is not expressed by endothelial cells of the CP (95) and although expressed in CP ependymal cells, it is primarily localized to the villi on the apical side of these cells (96).

**Figure 1:**
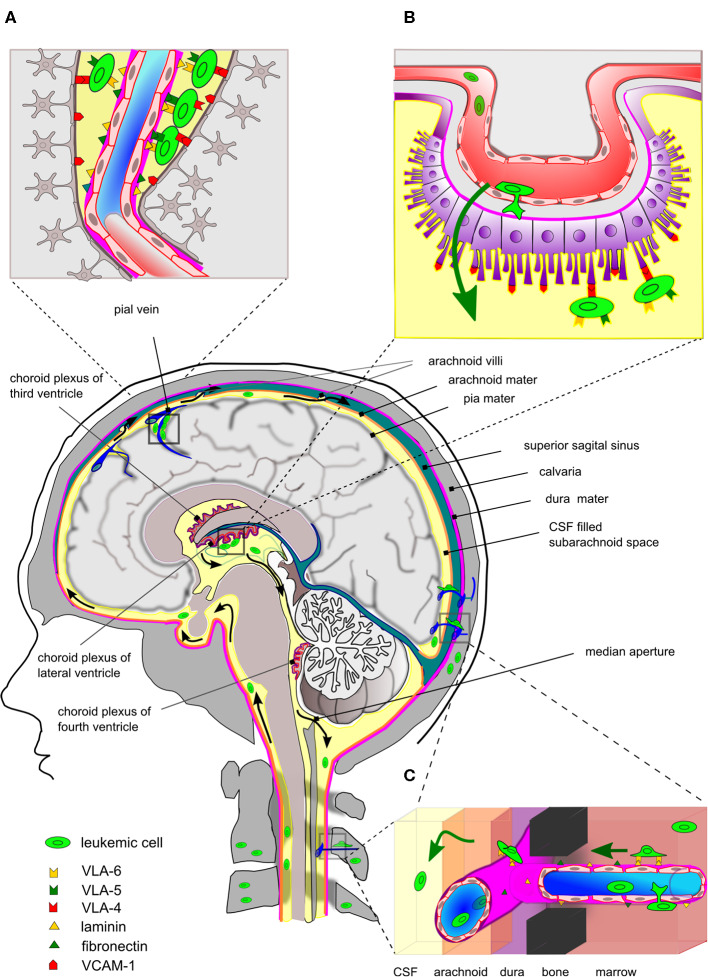
Entry routes and localization of ALL cells within the CNS. Simplistic drawing highlighting main entry routes and localization of ALL cells within the central nervous system and the proposed contribution of specific integrin-ligand interactions. **(A)** Leukemic cells are commonly found within the superficial arachnoid or seen pinched in the perivascular space around the post-capillary venules. At these sites, integrins may be involved in the binding to basal membrane proteins or VCAM-1 expressed by astrocytic feet processes. **(B)** The blood-cerebrospinal fluid barrier (BCSFB) is located at the choroid plexus (CP) located in the brain ventricles. The BCSFB is proposed as a main entry site for ALL cells into the CSF. The ALL cells in BM egress to peripheral blood circulation and subsequently arrive at microvessels underneath the CP, cross the layers of fenestrated endothelium and CP epithelial cells and can be seen adhering to VCAM-1 positive villi on the apical side. The latter suggests the involvement of integrins, such as VLA-4 (α4:β1). **(C)** Leukemic cells in the skull/vertebral bone marrow may migrate on the abluminal side of the endothelial cells of small vessels, thereby traversing through channels in compact bone and subsequently enter the meningeal space. This process may involve integrins binding basal membrane proteins as found for integrin α6 binding to laminin. For clarity, the drawing is disproportionate and anatomical details have been omitted. Black arrows indicate flow of CSF and green arrows indicate movement of leukemic blasts.

ALL has a marked tendency to disseminate to the CNS with T-ALL showing a higher incidence of CNS relapse than BCP-ALL (13, 17). Contrary to solid cancer metastatic cells, which invade the brain parenchyma, disseminated ALL is typically isolated to the leptomeninges. Early studies demonstrated that of 126 brains from autopsied leukemia patients, 42% had leukemic infiltration confined to the superficial/perivascular arachnoid while only 13% had signs of leukemic blasts within the brain parenchyma (97). Later studies, using xenograft mouse models, have shown that the ability to enter the CNS is a generic property of human ALL and not the result of rare selection of particular clones. It is difficult to ascertain the degree to which mouse-man differences contribute to the outcome of such xenotransplantation experiments. However, the histopathology accompanying the studies by Williams et al. showed striking similarities to the findings in patients, including leukemic infiltration around the dural venous sinuses (Figure 1A) and CP but not of brain parenchyma (19). Collectively, these data argue that leukemic blasts primarily transit the BCSFB rather than the BBB, similar to normal immune cells during immune surveillance in healthy brains (Figure 1B). *In vitro* culture models mimicking BCSFB have been developed based on rodent CP epithelial cells immortalized by SV40 large T antigen and immortalized CP epithelial cells from a human papilloma patient. Using such models, it was formally demonstrated that T-ALL and BCP-ALL cell lines can cross monolayers of CP epithelial cells in response to CSF-borne chemokines (98–100). In contrast, investigations of leukemic transmigration across BBB suggest that this form of crossing requires expression of particular adhesion molecules, not ubiquitously expressed by ALL cells (101), and only occurs secondary to endothelial activation by factors secreted from leukemic blasts or treatment-related neurotoxicity (102, 103).

As for normal lymphocytes (21), leukemic cells express most if not all integrins (79, 104). This provides ample possibilities for adhesion to the basal membranes of EC as well as ICAMs and VCAM-1 expressed on the surface of astrocytic foot processes (105), when leukemic cells are pinched in the perivascular space of post-capillary venules (Figure 1A). In addition, the integrin repertoire of leukemic cells would also allow adhesion to ICAMs and VCAM-1 on the apical side of the CP epithelial cells after traversing the BCSFB (96) (Figure 1B). Recently, Yao et al. proposed a non-hematogenous route into the CNS where leukemic blasts use integrin α6 to migrate on the laminin that is part of basal membranes on the abluminal site of emissary vessels (20) (Figure 1C). Such vessels connect vertebral or calvarial BM and the subarachnoid space of the spine and brain, respectively, and represent pathways employed by neural progenitors during neural development (106, 107). Yao et al. used a xenograft model based on Nalm-6 cells and primary human ALL cells combined with detailed histological examination and intravital fluorescent microscopy to provide compelling evidence for the migration of leukemic blasts on the outside of vessels. Their focus on integrin α6 resulted from the use of the PI3Kδ inhibitor GS-649443, which not only reduced CNS involvement in their xenograft model but also caused a reduction in *ITGA6* mRNA levels (20). However, blocking antibodies to α6 reduced but did not abolish CSF involvement in Nalm-6 engrafted mice (20) and therefore, the question remains whether α6 is actually alone in facilitating this form of non-hematogenous dissemination. In the work by Scharff et al., dissemination of BCP-ALL to the CSF was negatively correlated to surface α6/CD49f in clear contradiction of the results of Yao et al. Instead, a significant association was found between blasts in CSF and ITGA5 mRNA levels (79). Possibly, man-mouse differences could explain different outcomes in the mouse xenograft model used by Yao et al. and patient material investigated by Scharff et al. Furthermore, the latter work correlated the blast count in CSF with the leukemic integrin expression in BM samples, which may have overlooked subpopulations of ITGA6-expressing blasts that are actively engaged in adhesion and migration on the abluminal side of vessels connecting BM and CNS. With respect to ITGA5, functional studies are warranted to determine whether ALL blasts employ α5:β1 to migrate on the outside of vessels as proposed by Yao et al. (20).

Only few studies have addressed the role of the CNS microenvironment in conferring chemoresistance to leukemic cells and its link to integrin expression. Akers et al. used co-culture models to show that astrocytes, CP epithelial cells and meningeal cells increased the resistance of four ALL cell lines to cytarabine, dexamethasone and methotrexate commonly included in prophylactic regimens (108). Similarly, Gaynes et al. (81) found that CP epithelial cells conferred resistance to Nalm-6 cells to cytarabine and methotrexate. Of note, this study also investigated the differential impact of CNS and BM microenvironments on the transcriptome, finding that *ITGA6* was downregulated in Nalm-6 cells isolated from CNS compared to BM (81). The latter argues against the role of integrin α6/CD49f in facilitating the CNS involvement proposed by the work of Yao et al. (20), also based on Nalm-6 cells (20). The transcriptomic profile provided by the Gaynes et al. highlights the impact of microenvironment on the expression of integrins. Furthermore, the recent work of Jonart et al. showed that reducing leukemia-meningeal adhesion with Me6TREN (Tris[2-(dimethylamino)ethyl]amine) not only reduced leukemia chemoresistance but also the expression levels of several genes including *VCAM-1* (82). Since the expression levels of integrins and their ligands are influenced by microenvironment and adhesion, caution is warranted in the interpretation of expression studies associating expression of integrins with chemoresistance or tissue distribution.

## Perspectives

Although the current therapeutic regimens in ALL yield a 5-years overall survival of around 90%, 10–15% of patients continue to experience relapse (1). As illustrated in this review, compelling evidence exists for key roles of integrins in ALL cell survival, chemoresistance and CNS colonization, albeit clinical studies of integrins as therapeutic targets in ALL, and CNS involvement in particular, remain scarce. A wide variety of anti-integrin drugs are in clinical evaluation, among which a few are in clinical use for other diseases (8, 109), but at present, no integrin-targeted drugs have entered clinical trials in the treatment of CNS disseminated ALL. The integrin α4-inhibitor Natalizumab, has shown promising effect on ALL survival in mouse models in combination with chemotherapy (74, 87), but even though Natalizumab is known to prevent immune cell entry into the CNS in multiple sclerosis (94), it remains unclear whether it could also reduce CNS relapse frequency in ALL. Moreover, the risk of progressive multifocal leukoencephalopathy makes the compound potentially unsuitable for ALL patients (110). In general, integrins play important roles in normal immune cell functions, both inside and outside the CNS, making the risk of side effects of integrin-targeted therapy a real concern. Accordingly, further studies on the therapeutic role of integrins in ALL with CNS colonization are warranted.

In particular, ALL entry routes into the CNS need to be further elucidated. Recent, intriguing studies have suggested direct ALL/immune cell entry from calvarial bone marrow to the subarachnoid space along small vessels penetrating compact skull bone (20, 111), through specific interactions of adhesion molecules (20) and possibly driven by chemotactic signals (112). In support of this concept are post-mortem studies of ALL patients showing the meninges as a predilection site for ALL cells (97), as well as studies showing calvarial BM involvement as a frequent feature in ALL (113), but there is a clear need for further evidence on this matter. Unraveling such details of ALL cell CNS-entry mechanisms is a prerequisite for accurate and effective targeting of the integrins involved as a possible addition to the standard CNS prophylaxis in order to prevent CNS involvement and relapse of ALL.

## Author Contributions

All authors listed have made a substantial, direct and intellectual contribution to the work, and approved it for publication.

## Conflict of Interest

The authors declare that the research was conducted in the absence of any commercial or financial relationships that could be construed as a potential conflict of interest.
